# Brachiocephalic Vein and Superior Vena Cava Reconstruction with a Superficial Femoral Vein Graft

**DOI:** 10.21470/1678-9741-2020-0728

**Published:** 2022

**Authors:** Andres M Palacio, Maria A Medina, Diego H Marquez, Jaime Camacho, Albert F Guerrero

**Affiliations:** 1 Department of Cardiovascular Surgery, Fundación Cardioinfantil, Bogotá, Colombia.

**Keywords:** Superior Vena Cava Syndrome, Superior Vena Cava, Brachiocephalic Veins, Superficial Vena Cava Syndrome, Vascular Reconstruction

## Abstract

Superior vena cava syndrome (SVCS) is an entity that has become more frequent due to the increasing use of indwelling central venous catheters. Surgical management is considered in patients with extensive venous thrombosis and when endovascular therapy is not feasible. The use of superficial femoral vein is an excellent technique for reconstruction of the brachiocephalic vein and superior vena cava (SVC) in cases with benign and malignant etiologies. We describe two cases of SVCS that were managed surgically at our institution with replacement of the SVC and brachiocephalic veins with a superficial femoral vein graft technique.

**Table:** Abbreviations, acronyms & symbols

CI	= Confidence interval
CT	= Computed tomography
CTA	= Computed tomography angiography
SFV	= Superficial femoral vein
SVC	= Superior vena cava
SVCS	= Superior vena cava syndrome

## INTRODUCTION

The first description of superior vena cava syndrome (SVCS) was published by William Hunter, in 1757, occurring in a patient with a mycotic aortic aneurysm^[1]^.

About 17,000 patients develop symptoms of venous congestion of the head and neck due to occlusion of the superior vena cava (SVC) and brachiocephalic veins in the United States of America every year^[2,3]^. Malignancy is the leading cause of SVCS in 60-80% of the cases^[2,4]^; however, the incidence of nonmalignant causes has been increasing secondary to the expanded use of indwelling central venous catheters and cardiac pacemaker wires^[2,3,5]^. Up to 40% of all patients with central venous lines develop thrombosis and 1-14% develop SVCS^[6]^.

Patients can be managed with conservative measures, endovascular therapy, or open surgical approach^[2]^. Percutaneous transluminal angioplasty and stenting are the most frequently used strategies for SVCS of malignant etiology with concomitant thrombolysis therapy in selected cases^[6]^.

Surgical reconstruction is usually considered in patients with extensive venous thrombosis not suitable for endovascular treatment, also in those with failed percutaneous interventions and refractory symptoms^[2,3,5]^. There are several types of conduits for SVC and brachiocephalic vein reconstruction, from autologous grafts — such as superficial femoral vein (SFV), spiral saphenous vein, and pericardial tubular conduits — to expanded polytetrafluoroethylene prosthetic grafts^[6]^.

The first report of SVC bypass was published in 1951 by Klassen et al.^[7]^. The SFV was one of the first bypass conduits used for reconstruction of SVC^[6]^.

We describe a technique of SVC and brachiocephalic veins reconstruction with an SFV graft in two patients who developed SVCS and were managed at our institution. The Fundación Cardioinfantil ethics committee approved the study (Minutes Nº25-2021). All the procedures in this study were in accordance with the 1975 Helsinki Declaration, updated in 2013.

### Case Nº 1

**Fig. 1:**
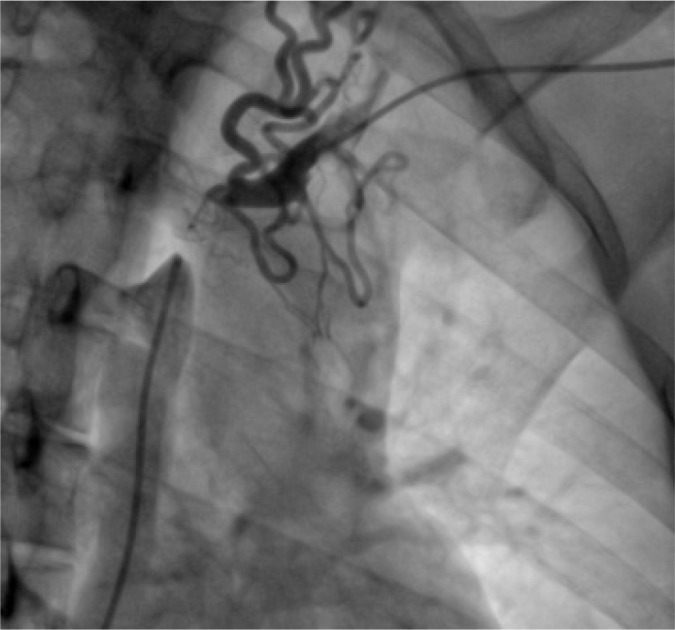
Invasive angiography showing occlusion of the superior vena cava and brachiocephalic vein with collateral circulation.

A 35-year-old male patient with a history of chronic autoimmune renal failure on hemodialysis for four years required placement of several jugular indwelling catheters and developed SVCS in the last two years. At an outside hospital, they failed doing endovascular therapy. The patient was transferred to our institution looking for definitive treatment. Computed tomography (CT) and invasive angiography imaging confirmed diagnosis (Figure 1). A new attempt of percutaneous management was done; nevertheless, complete occlusion of the brachiocephalic vein and SVC was found, then surgical treatment was decided.

**Fig. 2:**
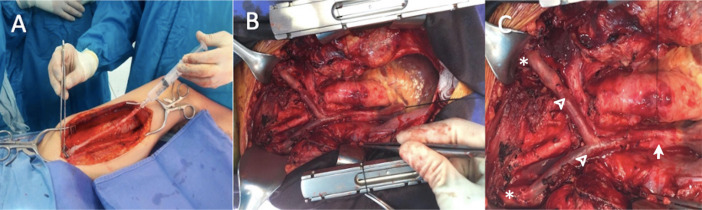
Intraoperative images of a bilateral internal jugular vein to superior vena cava (SVC) bypass with a reversed left femoral vein autograft interposition. (A) Left superficial femoral vein harvesting, (B) and (C) complete bypass in situ (asterisk = right and left internal jugular veins; arrowhead = superficial femoral vein graft; arrow = SVC).

The patient underwent bilateral internal jugular vein to SVC bypass surgery with interposition of a reversed SFV autograft harvested from the left lower limb. Through a medium sternotomy extending to the neck, the thrombosed SVC and brachiocephalic veins were identified, the graft was placed with an end-to-side proximal anastomosis to the SVC and an end-to-side distal anastomosis to the right and left internal jugular veins with an end-to-side neo-confluent, this was performed without using cardiopulmonary bypass (Figure 2). The patient was taken to the surgical intensive care unit, extubated after six hours, and transferred to a general ward on postoperative day one. He was discharged after five days on full oral anticoagulation and a comprehensive rehabilitation plan.

**Fig. 3:**
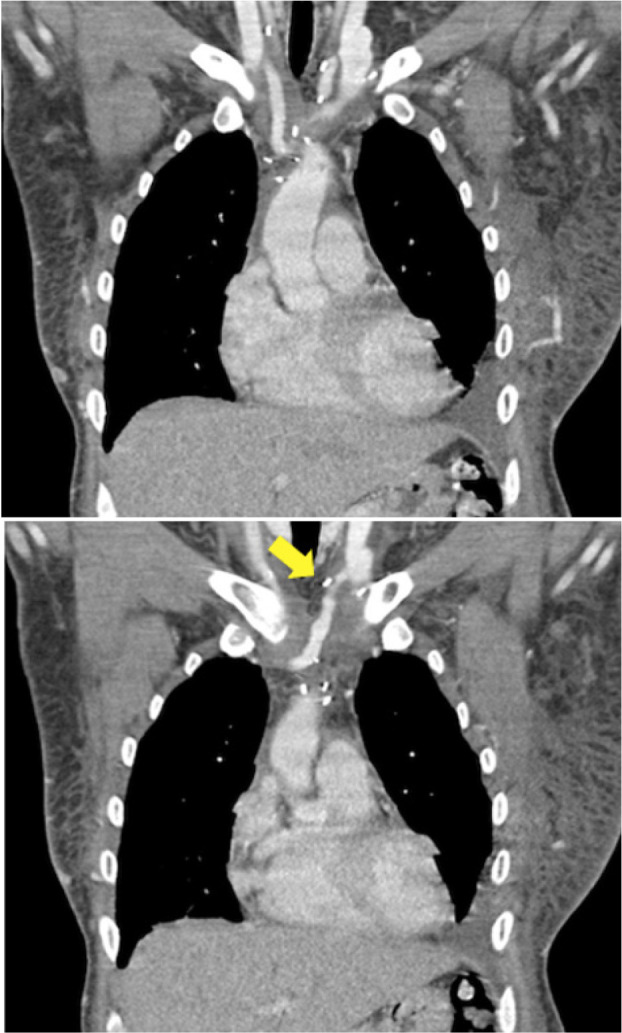
One-year follow-up venous computed tomography angiography images demonstrating complete patency of the right jugular-cava confluent and a 20% reduction in the patency of the left branch shunt (arrow).

Follow-up was conducted with venous phase computed tomography angiography (CTA) (Figure 3). The patient continued management with full oral anticoagulation and lymphatic drainage to reduce pectoral and left upper limb edema. After a three-year follow-up, the patient is asymptomatic, fully resolved the SVCS, and has not required any additional interventions.

### Case Nº 2

**Fig. 4:**
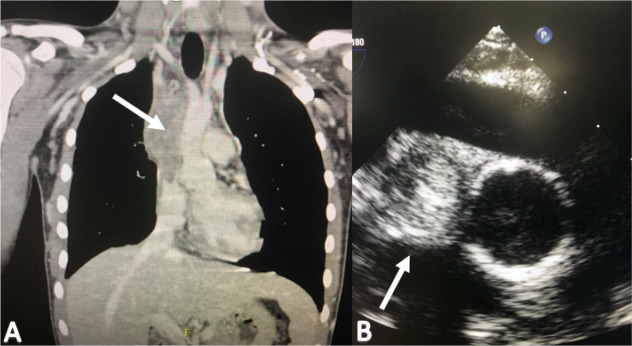
(A) Computed tomography angiography showing thrombosis of the superior vena cava (arrow). (B) Transesophageal echocardiogram images showing 90% occlusion of the superior vena cava circumference (arrow).

A 17-year-old male patient consulted with us because of a one-year history of facial and right upper limb edema, which were confirmed on physical exam. The patient had had a resection of a mature teratoma two years before and received complementary chemotherapy and percutaneous embolization. He arrived at our clinic with a CT scan showing SVC and brachiocephalic veins obstruction secondary to thrombosis, that was also observed on transesophageal echocardiogram (Figure 4), for which two unsuccessful angioplasties were intended at another institution.

**Fig. 5:**
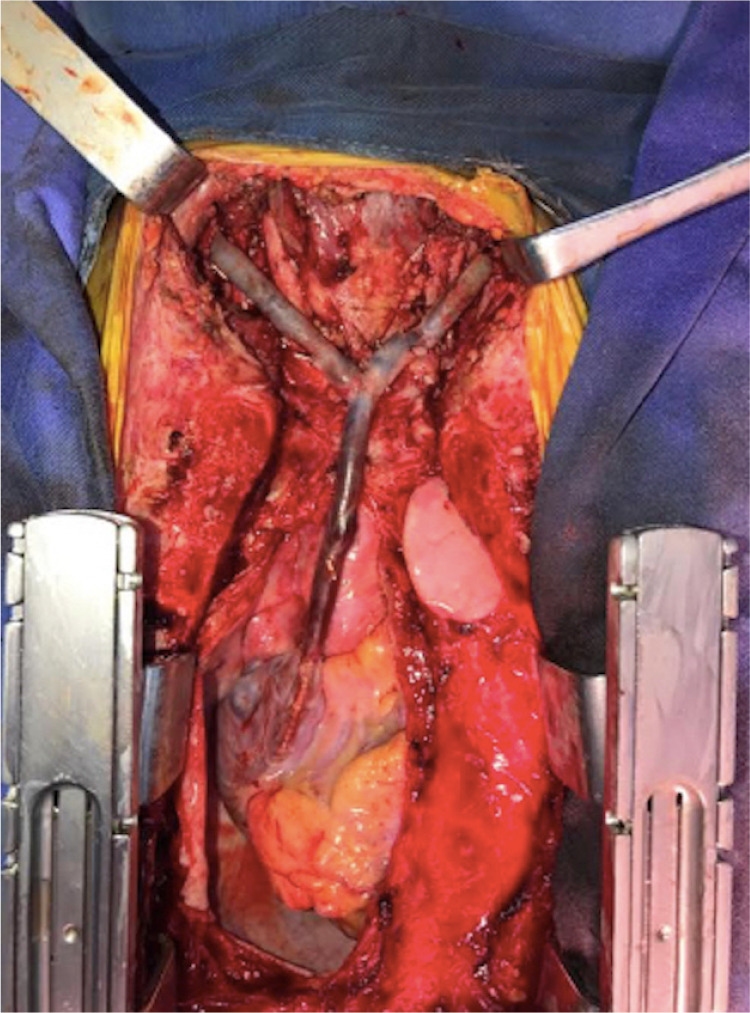
Bilateral internal jugular vein to right atrium bypass with a reversed superficial femoral vein graft.

We decided to perform a surgical treatment with a bilateral internal jugular vein to right atrium bypass procedure using a reversed SFV autograft harvested from the left lower limb without cardiopulmonary bypass (Figure 5). Surgical findings were severe fibrosis of the anterior mediastinum and thrombosis of the SVC and brachiocephalic veins. There was no evidence of tumoral relapse in the operative field. The histopathologic study revealed thrombus formation, without any tumoral cells remaining.

Tracheal extubation was successfully accomplished in the operating room. The patient did not require any vasoactive drugs at the intensive care unit, was transferred to a general ward on postoperative day two, and was discharged home after four days of surgery. The patient was asymptomatic for a year; nevertheless, follow-up CT scan revealed a teratoma recurrence and he died one year later.

## DISCUSSION

Although both open and endovascular treatments of SVCS have shown good results, Sfyroeras et al.^[3]^ made the largest published review of 13 studies, including 87 patients who underwent open surgical repair with better long-term patency rates compared to 136 patients who received endovascular therapy.

Surgical replacement of the SVC with autologous vein grafts has been shown to have excellent long-term results in terms of patency in contrast to synthetic grafts^[5,8]^. Doty et al.^[9,10]^ described 16 patients who underwent SVC bypass with spiral saphenous vein graft for SVC obstruction secondary to benign disease; they observed the long-term results and reported 14 out of 16 patients with 87.5% grafts remaining patent at a mean of 10.9 years of follow-up and all patients, except for one, were free from SVCS. The longest follow-up lasted for 23 years and eight months.

SFV grafts are a versatile type of conduits that have been used for arterial and venous reconstruction. Brahmanandam et al.^[11]^ reported their experience in 42 patients using SFV grafts, showing secondary patency rates of 100% at 30 days, 97.1% at one year, 89% at three years of follow-up (95% confidence interval [CI], 74.2-100), and survival rates over 86% at three years of follow-up (95% CI, 75.3-98.3).

Among the advantages of using SFV grafts we can find: the SFV does not require to be modified prior to use, it is similar in size to the internal jugular vein, it has an average large caliber and length, also it can be easily harvested with approximately 30-cm graft available from the confluence with the profunda femoris to the above-knee popliteal vein^[2]^. The SFV has a theoretical antithrombogenic benefit over spiral grafts, because it lacks long suture lines, requires less incisions, and does not demand so much time for graft construction^[2,6]^.

In our cases, we were able to see a benign condition secondary to jugular indwelling catheters thrombosis where the brachiocephalic veins and SVC reconstruction was achieved uneventfully with complete resolution of the symptoms. Even though the follow-up period is still short, the graft has shown a satisfactory behavior. Unfortunately, the second case had a relapse of a malignant teratoma almost two years after successful SVC reconstruction which precluded the possibility of determining the long-term patency of the graft.

## CONCLUSION

Using an SFV graft to treat severe SVCS is an excellent choice given the good long-term patency rates and the fact that grafts can be easily harvested with very low morbidity. In patients with benign disease, it is preferable to use an autologous vein graft, especially because these patients have a longer life expectancy.
